# Impairment of Endothelial-Myocardial Interaction Increases the Susceptibility of Cardiomyocytes to Ischemia/Reperfusion Injury

**DOI:** 10.1371/journal.pone.0070088

**Published:** 2013-07-22

**Authors:** Thorsten M. Leucker, Zhi-Dong Ge, Jesse Procknow, Yanan Liu, Yang Shi, Martin Bienengraeber, David C. Warltier, Judy R. Kersten

**Affiliations:** 1 Department of Anesthesiology, Medical College of Wisconsin, Milwaukee, Wisconsin, United States of America; 2 Department of Surgery, Medical College of Wisconsin, Milwaukee, Wisconsin, United States of America; 3 Deparment of Pharmacology and Toxicology, Medical College of Wisconsin, Milwaukee, Wisconsin, United States of America; Virginia Commonwealth University Medical Center, United States of America

## Abstract

Endothelial-myocardial interactions may be critically important for ischemia/reperfusion injury. Tetrahydrobiopterin (BH_4_) is a required cofactor for nitric oxide (NO) production by endothelial NO synthase (eNOS). Hyperglycemia (HG) leads to significant increases in oxidative stress, oxidizing BH_4_ to enzymatically incompetent dihydrobiopterin. How alterations in endothelial BH_4_ content impact myocardial ischemia/reperfusion injury remains elusive. The aim of this study was to examine the effect of endothelial-myocardial interaction on ischemia/reperfusion injury, with an emphasis on the role of endothelial BH_4_ content. Langendorff-perfused mouse hearts were treated by triton X-100 to produce endothelial dysfunction and subsequently subjected to 30 min of ischemia followed by 2 h of reperfusion. The recovery of left ventricular systolic and diastolic function during reperfusion was impaired in triton X-100 treated hearts compared with vehicle-treated hearts. Cardiomyocytes (CMs) were co-cultured with endothelial cells (ECs) and subsequently subjected to 2 h of hypoxia followed by 2 h of reoxygenation. Addition of ECs to CMs at a ratio of 1∶3 significantly increased NO production and decreased lactate dehydrogenase activity compared with CMs alone. This EC-derived protection was abolished by HG. The addition of 100 µM sepiapterin (a BH_4_ precursor) or overexpression of GTP cyclohydrolase 1 (the rate-limiting enzyme for BH_4_ biosynthesis) in ECs by gene trasfer enhanced endothelial BH_4_ levels, the ratio of eNOS dimer/monomer, eNOS phosphorylation, and NO production and decreased lactate dehydrogenase activity in the presence of HG. These results demonstrate that increased BH_4_ content in ECs by either pharmacological or genetic approaches reduces myocardial damage during hypoxia/reoxygenation in the presence of HG. Maintaining sufficient endothelial BH_4_ is crucial for cardioprotection against hypoxia/reoxygenation injury.

## Introduction

Hyperglycemia (HG) is common in patients suffering from acute coronary syndrome and is associated with poor prognosis and increased mortality rates. [1], [2] A series of interconnected biochemical changes initiated by HG have been documented to directly affect vascular and myocardial function resulting in the development of cardiovascular complications.[3]–[5] Endothelial dysfunction, characterized by a loss of nitric oxide (NO) bioactivity, is generally regarded as one of the most important cellular events accounting for the adverse effects of HG on the cardiovascular system.[6]–[10] It remains elusive how endothelial dysfunction caused by acute HG impacts the susceptibility of cardiomyocytes to ischemia/reperfusion (I/R) injury.

Endothelial nitric oxide synthase (eNOS) is highly expressed in the endothelial cells of the cardiac vasculature. [11], [12] eNOS enzyme consists of a heme-containing oxygenase domain that binds the essential co-factor tetrahydrobiopterin (BH_4_), molecular oxygen, and the substrate L-arginine; and a reductase domain that transfers electrons from NADPH to FAD and FMN. [13] In the presence of BH_4_ and L-arginine, heme and oxygen reduction are coupled to the synthesis of NO. In addition to its potent vasodilatory effect, eNOS-derived NO has been demonstrated to be cardioprotective.[14]–[19] However, HG leads to significant increases in oxidative stress, oxidizing BH_4_ to enzymatically incompetent dihydrobiopterin, which competes with BH_4_ for eNOS binding.[7], [20]–[23] When BH_4_ levels are inadequate, oxygen reduction by eNOS is uncoupled to L-arginine oxidation, resulting in the generation of the cardiotoxic mediator superoxide rather than NO. [24], [25] Therefore, in the present study, we tested the hypothesis that endothelial dysfunction exacerbates myocardial I/R injury, and that increased coronary vascular endothelial BH_4_ content by pharmacological and genetic approaches protects the cardiomyocyte (CM) against I/R injury during HG.

## Materials and Methods

### 1. Animals

Male C57BL/6 mice (weight: 26.5±0.5 g; age: 9–12 weeks) and pregnant female Wistar rats (age: 9–12 weeks) were purchased from Jackson Laboratory (Bar Harbor, ME, USA) and Charles River Laboratories International, Inc. (Wilmington, MA, USA), respectively. The animals were kept on a 12-h light-dark cycle in a temperature-controlled room. All experimental procedures used in this study were approved by the Animal Care and Use Committee of the Medical College of Wisconsin and conformed to the Guide for the Care and Use of laboratory Animals (NIH Publication No. 85-23, revised 1996).

### 2. Langendorff Perfusion of Mouse Heart

**Figure 1:**
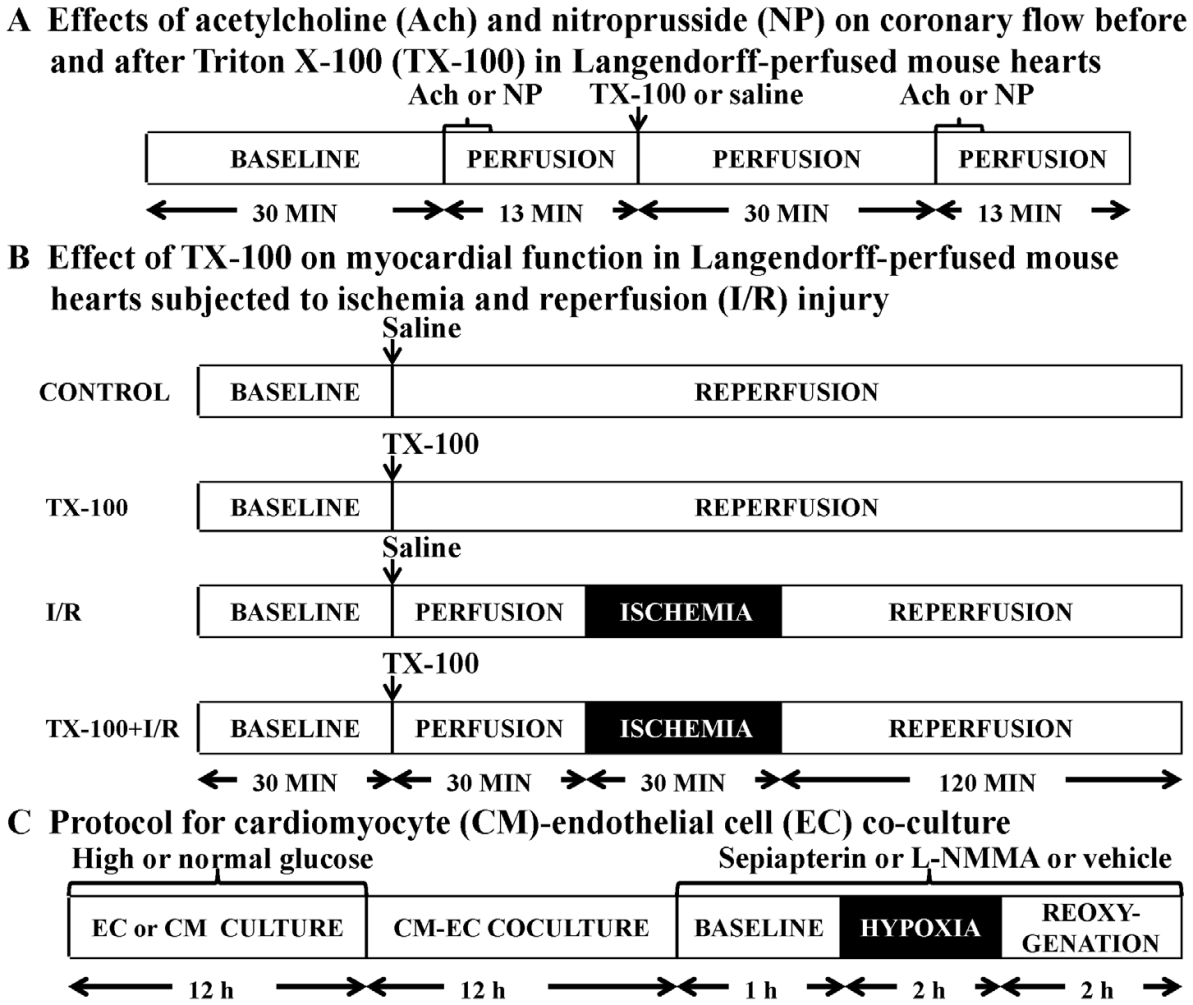
Schematic representation of the experimental protocols. (A) Effects of Ach and NP on coronary flow before and after the administration of TX-100 in Langendorff-perfused mouse hearts; (B) effects of TX-100 on myocardial function in Langendorff-perfused mouse hearts subjected to ischemia and reperfusion injury; (C) effects of high glucose, L-NAME, sepiapterin, and GTP cyclohydrolase 1 overexpression in endothelial cells on hypoxia and reoxygenation injury in CM-EC co-culture.

We have previously described Langendorff perfusion of mouse hearts.[22], [26]–[28] Briefly, once excised, the hearts were mounted on a Langendorff apparatus and perfused retrogradely through the aorta at a constant pressure of 80 mmHg with Krebs-Henseleit buffer containing (in mM) NaCl 118, NaHCO_3_ 25, KCl 4.7, MgCl_2_ 1.2, CaCl_2_ 2.5, KH_2_PO_4_ 1.2, EDTA 0.5, and glucose 11. The buffer was continuously bubbled with a mixture of 95% oxygen/5% carbon dioxide via an in-line filter (5 µm pore size). A fluid-filled plastic balloon was inserted into the chamber of the left ventricle (LV) via the mitral valve, and connected to a pressure transducer for continuous measurement of LV pressure. The hearts were immersed in perfusate maintained at 37.2±0.3°C, and the balloon was inflated to a diastolic pressure of ∼5 to 10 mmHg. Coronary flow was monitored by an in-line flow probe connected to a flow meter (Transonics Systems Inc., Ithaca, NY, USA). The LV pressure signal was monitored to obtain heart rate and LV dP/dt. The LV developed pressure (LVDP) was calculated as the difference between systolic and end-diastolic LV pressure. Global I/R was produced by cessation of perfusion followed by reperfusion at a designated time. Langendorff-perfused hearts were used in two protocols (Figure 1).

#### Protocol A

Previous studies demonstrated that the bolus injection of 1∶200 triton X-100 (TX-100, Sigma-Aldrich, St. Louis, MO, USA) at 1% of coronary flow into the coronaries in Langendorff-perfused rat and rabbit hearts rendered the coronary endothelium dysfunctional.[29]–[31] However, equivalent amounts of TX-100 caused 7 out of 10 hearts to suffer irreversible contractile failure in Langendorff-perfused mouse hearts. Pilot experiments showed that the bolus administration of 1∶200 TX-100 at 0.3% of coronary flow did not cause myocardial contractile failure. Thus, we determined the effect of the endothelium-dependent vasodilator acetylcholine (Ach, Sigma-Aldrich) and endothelium-independent vasodilator nitroprusside (NP, Sigma-Aldrich) on coronary flow before and after the bolus injection of 1∶200 TX-100 at 0.3% of coronary flow in Langendorff-perfused C57BL/6 mouse hearts (Figure 1A). All hearts were perfused for 30 min for stabilization, and baseline coronary flow was recorded. Hearts were then randomly assigned to 4 groups (n = 5 hearts/group). In Group 1 and 2, the hearts were perfused with 0.5 µM Ach for 3 min followed by washout for 10 min. After a bolus injection of TX-100 or an equivalent amount of saline followed by washout for 30 min, the hearts were perfused with 0.5 µM Ach for 3 min followed by washout for 10 min again. In Group 3 and 4, the hearts were perfused with 30 µM NP for 3 min followed by washout for 10 min. After a bolus injection of TX-100 or saline followed by washout for 30 min, the hearts were perfused with 30 µM NP for 3 min followed by washout for 10 min.

#### Protocol B

To evaluate the effects of coronary endothelial dysfunction on cardiac function (Figure 1B), C57BL/6 mice were randomly assigned to 1 of the following 4 groups (n = 6–8 mice/group): (1) control; (2) TX-100; (3) I/R; and (4) I/R+TX-100. After stabilization for 30 min, the hearts in I/R and I/R+TX-100 groups were subjected to 30 min of global ischemia and 2 h of reperfusion with or without bolus injection of 1∶200 TX-100 at 0.3% of coronary flow. The hearts in control and TX-100 groups were not subjected to ischemia. LVDP, +dP/dt (maximum rate of increase of LVDP), and –dP/dt (maximum rate of decrease of LVDP) at baseline, 10, 20, and 30 min post-administration of TX-100, and 10, 30, 60, 90, and 120 min after reperfusion were determined.

### 3. Cell Culture

Endothelial cells (ECs) isolated from coronary arteries of healthy subjects (Cell Applications, San Diego, CA, USA) were cultured in MesoEndo cell growth medium (Cell Applications) at 37°C and used between 4th and 6th passages when approximately 70–80% confluent. CMs were isolated from hearts of one-day-old Wistar rats by repeating enzyme digestion (0.15 mg/ml collagenase II and 0.52 mg/ml pancreatin (Sigma-Aldrich), as previously described. [32] CMs were used 3–7 days after isolation when demonstrating rhythmic contractions.

### 4. EC-CM Co-culture

ECs and/or CMs were cultured in media containing 5.5 mM (normal glucose concentration, NG) or 20.0 mM glucose (high glucose concentration, HG) for 12 h. For co-culture, ECs were mixed and cultured with CMs at distinct ratios for 12 h, depending on the design of the experiment. [33] Culture medium was then replaced and the cells were exposed to 2 h of hypoxia (0.1% O_2,_ Biospherix hypoxia chamber, Lacona, NY, USA) in glucose-free medium followed by 2 h of reoxygenation (Figure 1, protocol C). In some experiments, cells were treated with 1 mM L-N^G^-monomethyl arginine (L-NMMA, Cayman Chemical Company, Ann Arbor, MI, USA), a non-specific NOS inhibitor. To investigate the effect of BH_4_ in ECs, 100 µM sepiapterin (SEP, Sigma-Aldrich) was added to co-cultured cells as substrate for the synthesis of BH_4_ during 60 min of baseline and the period of hypoxia/reoxygenation (H/R). The release of lactate dehydrogenase (LDH) into the culture medium as a marker of cell damage was quantified with a commercially available kit (Genzyme Diagnostics, Cambridge, MA, USA).

### 5. Overexpression of GTPCH-1 Gene in ECs

GTPCH-1 was amplified from pCMV-sport6 (cDNA, Open Biosystems, Waltham, MA, USA) using primers 5′- AGA CAC CGA CTC TAG - GTA TAC - GCC ACC - ATG GAG AAG CCG CGG-3′ and 5′- CCG CTT TAC TTG TAC - TCA AGC GTA ATC TGG AAC ATC GTA TGG GTA - GTA TAC - GCT CAG CTC CTG ATT AGT GTG-3′ (C- terminal HA tag) and 5′- AGA CAC CGA CTC TAG - GCC ACC - ATG GAG TAC CCA TAC GAT GTT CCA GAT TAC GCT - GTA TAC - ATG GAG AAG CCG CGG-3′ and 5′- CCG CTT TAC TTG TAC - GTA TAC - TCA GCT CAG CTC CTG ATT AGT-3′ (N-terminal HA tag) and cloned into pLenti-CMV-GFP-Puro (#17448, Addgene, Cambridge, MA, USA) between the XbaI and Bsp1407I sites, using In-Fusion HD cloning system (Clontech, Mountain View, CA, USA). [34].

Lentivirus vectors were produced in roller bottles by co-transfection of 293T cells with 79 µg pLenti-CMV-CHA/NHA-GTPCH1, 52 µg psPAX2 packaging (#12260, Addgene), and 26 µg pMD2.G (VSV-G envelope, #12259, Addgene) using Polyethylenimine (Polysciences Warrington, PA, USA). Transfection medium was replaced with sodium butyrate-containing medium at 12–16 h post transfection, and virus-containing supernatant was harvested 24–32 h later (36–48 h post transfection). Supernatant was cleared, filtered through 0.22 µm PES and concentrated by centrifugation at 5000×g for 20 h. The virus pellet was resuspended in PBS and nutated for ∼3 h at 4°C, aliquoted, and frozen at −80°C. The viral titer was determined by quantitative PCR analysis of viral LTR-positive 293T cells. Pilot experiments using a GFP expressing lentiviral vector were performed to determine the optimal multiplicity of infection (MOI), which was found to be 3 (95–100% GFP-positive EC). ECs were infected in a 12-well plate by incubating with viral particles for 24 h followed by a viral washout.

### 6. Ozone Chemiluminescence

Nitrite concentration (an index of NO) was measured in the cell culture medium using a Sievers NO gas analyzer (Model 280, GE Analytical Instruments, Boulder, CO, USA), as previously described. [33], [35] Briefly, cell culture media (1 ml) from ECs, CMs, and EC-CM co-culture were collected at two time points (60 min at baseline and 2 h after reoxygenation) and immediately frozen in liquid nitrogen. Nitrite concentration was calculated after subtraction of background levels and normalized to total cell protein from cell lysates prepared as described for Western blotting.

### 7. BH_4_ Assay

BH_4_ was quantified by high performance liquid chromatography with electrochemical detection (ESA Biosciences CoulArray® system Model 542, Chelmsford, MA, USA), as previously described. [21] In brief, endothelial cell pellets were immediately lysed in 300 µl of 50 mM phosphate buffer (pH 2.6) containing 0.2 mM diethylenetriaminepentaacetic acid and 1 mM dithioerythritol (freshly added) by shearing cells with a 28-gauge tuberculin syringe. Samples were centrifuged (12000×*g*, 10 min, 4°C), and supernatants were filtered through a 10 kD molecular weight cutoff column (Millipore, Billerica, MA). One hundred eighty µl of the flow through was analyzed by using a Synergi Polar-RP column (Phenomex, Torrance, CA, USA) eluted with argon-saturated 50 mM phosphate buffer (pH 2.6). Multi-channel electrochemical detection was set between 0–600 mV. One channel was set at −250 mV to verify the reversibility of BH_4_ oxidative peak detection. Calibration curves were constructed by summation of peak areas collected at 0 and 150 mV for BH_4_. Intracellular BH_4_ concentrations were calculated using authentic BH_4_ as standard and normalized to cell protein concentrations.

### 8. Western Blot Analysis of eNOS

The expression of eNOS in cultured CMs and ECs at a ratio of 1∶3 was measured in three separate groups. Expression of eNOS homodimers and monomers was evaluated in Groups 1 and 2. In Group 1, the cells were divided into the following four subgroups (n = 4 cells dishes/group): NG, HG, HG+SEP, and HG+SEP+L-NMMA. The treatment of cells with HG, SEP, and L-NMMA was described in Section 2.4. In Group 2, cells were assigned to the following three subgroups (n = 4 cell dishes/group): NG, HG+ConVec (control vector), and HG+GTPCH-1 OE (GTPCH-1 overexpressing ECs). ECs in NG group were cultured for 12 h in normoglycemic medium prior to co-culture with CMs. ECs in HG+GTPCH-1OE group were infected with lentiviral vectors (as described in section 2.5) for 24 h, cultured for 24 h without lentiviral vectors in normoglycemic medium and for 12 h in hyperglycemic medium, and co-cultured with CMs. ECs in HG+ConVec were infected with the control vector (as described in section 2.5) for 24 h, cultured for 24 h without lentiviral vectors in normoglycemic medium and for 12 h in hyperglycemic medium, and co-cultured with CMs. All co-cultured cells underwent 2 h of hypoxia followed by 2 h of reoxygenation. To investigate eNOS homodimer formation in CM-EC co-culture, nonboiled cellular lysate was resolved by 6% SDS-PAGE at 4°C overnight, as previously described. [23] Membranes were incubated with a 1∶2000 dilution of mouse anti-eNOS monoclonal antibody (BD Transduction Laboratories, San Jose, CA, USA). The expression of total eNOS and phosphorylated eNOS (phos-eNOS) was examined in Group 3. The cells were assigned to the following five subgroups (n = 6 cell dishes/group): NG, HG, HG+SEP, HG+SEP+L-NMMA, and HG+GTPCH-1 OE. Total soluble protein from cell lysates was prepared as previously described. [21], [23] In brief, 50 µg of protein was resolved on a 7.5% SDS-polyacrylamide gel, proteins transferred to polyvinylidene fluoride membranes, and the membranes blocked in tris-buffered saline containing 5% milk. The membranes were incubated with primary antibodies against eNOS (Santa Cruz Biotechnologies, Santa Cruz, CA, USA) and phos-eNOS (Cell Signaling, Boston, MA, USA) overnight at 4°C, washed and then incubated with the appropriate secondary antibody. Immunoreactive bands were visualized by enhanced chemiluminescence followed by densitometric analysis using image acquisition and analysis software (Image J, NIH). β-actin (Abcam, Cambridge, MA, USA) was used to normalize the expression of eNOS and phosphorylated eNOS (phos-eNOS).

### 9. Statistical Analysis

All data are expressed as mean ± S.E.M. Statistical analysis was performed with one-way ANOVA followed by Bonferroni *post-hoc* test for multiple comparisons of multiple group means or with Student’s *t* test for comparisons between two groups. A value of P < 0.05 was considered statistically significant.

## Results

### 1. Impact of Coronary Endothelium on Myocardial I/R Injury

**Figure 2:**
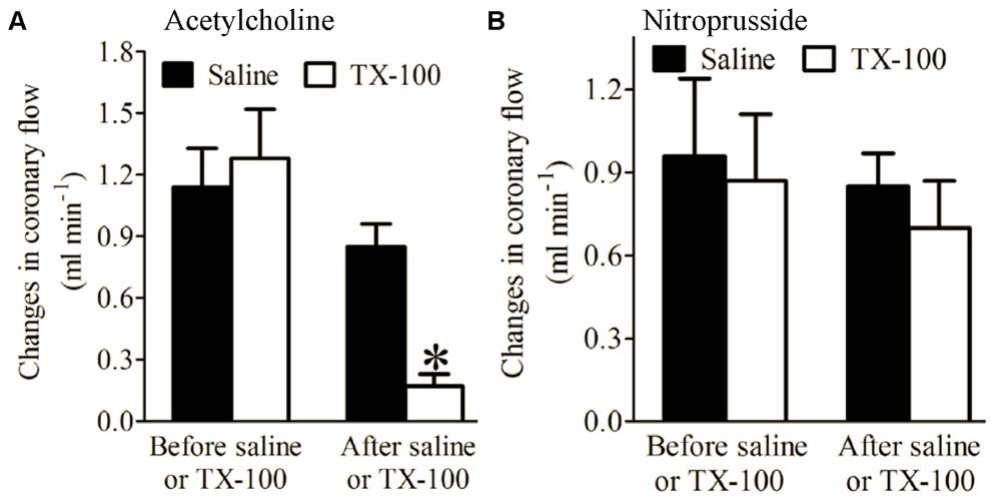
Triton X-100 (TX-100) decreased acetylcholine-induced, but did not alter nitroprusside-induced changes in coronary flow. (A) Acetylcholine-induced changes in coronary flow; (B) nitroprusside-induced changes in coronary flow. Langendorff-perfused hearts were stabilized for 30 min and perfused with acetylcholine or nitroprusside for 3 min followed by washout for 10 min both before and after a bolus injection of TX-100 or saline. *P < 0.05 vs. saline (n = 5 hearts/group).

There were no significant differences in baseline coronary flow of Langendorff-perfused hearts between groups. Ach and NP significantly increased coronary flow both before and after saline (control) injection (Figure 2). In contrast, after a bolus injection of 1∶200 TX-100 at 0.3% of coronary flow, Ach-induced increases in coronary flow were significantly diminished compared with the saline-treated group (n = 5 hearts/group, P < 0.05) (Figure 2A). However, there were no significant differences in NP-induced increases in coronary flow between TX-100 and saline-treated groups (Figure 2B).

**Figure 3:**
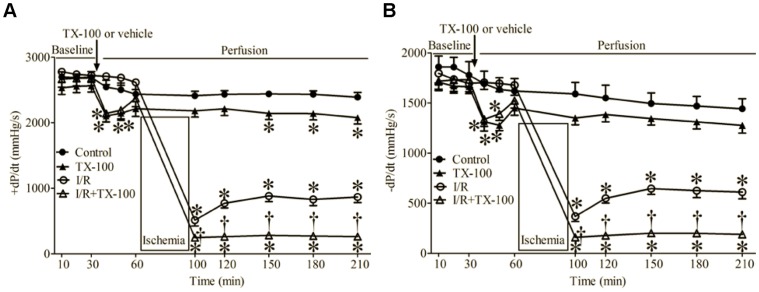
Triton X-100 (TX-100) attenuated the recovery of cardiac function during reperfusion in Langendorff-perfused hearts subjected to ischemia and reperfusion (I/R) injury. (A) +dP/dt (maximum rate of increase of LVDP); (B) -dP/dt (maximum rate of decrease of LVDP). Langendorff-perfused hearts were stabilized for 30 min and received a bolus injection of TX-100 or saline. Hearts in I/R and TX-100+I/R groups were subjected to ischemia followed by 2 h of reperfusion, whereas control and TX-100 groups did not undergo ischemia. *P < 0.05 vs. control; ^†^P < 0.05 vs. I/R (n = 6–8 hearts/group).

The effects of TX-100 on cardiac function in Langendorff-perfused hearts are shown in Figure 3. Baseline values of +dP/dt and -dP/dt were comparable among groups. Bolus injection of TX-100 caused significant decreases in +dP/dt and -dP/dt 10 and 20 min after TX-100. Ventricular function (+dP/dt and -dP/dt) was partially recovered after I/R in vehicle-perfused hearts. Treatment of hearts with TX-100 resulted in more profound decreases in +dP/dt and -dP/dt during reperfusion as compared with vehicle-perfused hearts.

### 2. EC-derived Protection of CMs from H/R Injury is Dependent on NO and Sensitive to Glucose

**Figure 4:**
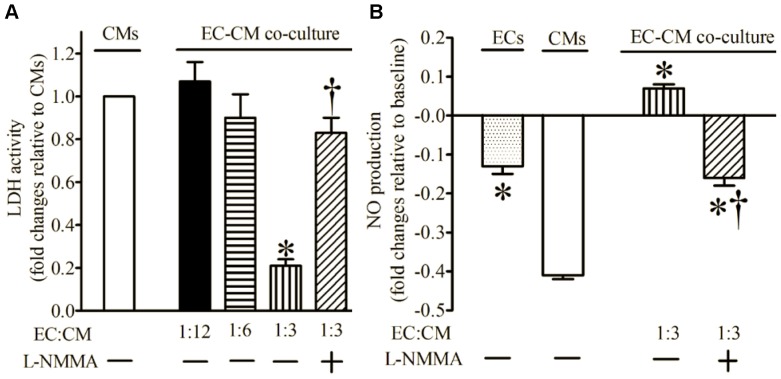
Co-culture of endothelial cells (ECs) and cardiomyocytes (CMs) decreased the activity of lactate dehydrogenase (LDH) and increased nitric oxide (NO) production following hypoxia and reoxygenation injury. (A) EC:CM ratio-dependent changes in LDH activity; (B) increased production of NO by ECs in co-culture. All cells were subjected to 2 h of hypoxia followed by 2 h of reoxygenation. LDH activity was expressed as fold changes relative to CMs, and NO as fold changes relative to baseline. L-NMMA = L-N^G^-monomethyl arginine. *P < 0.05 vs. CMs; ^†^P < 0.05 vs. EC-CM (1∶3) co-culture (n = 6 dishes/group).

**Figure 5:**
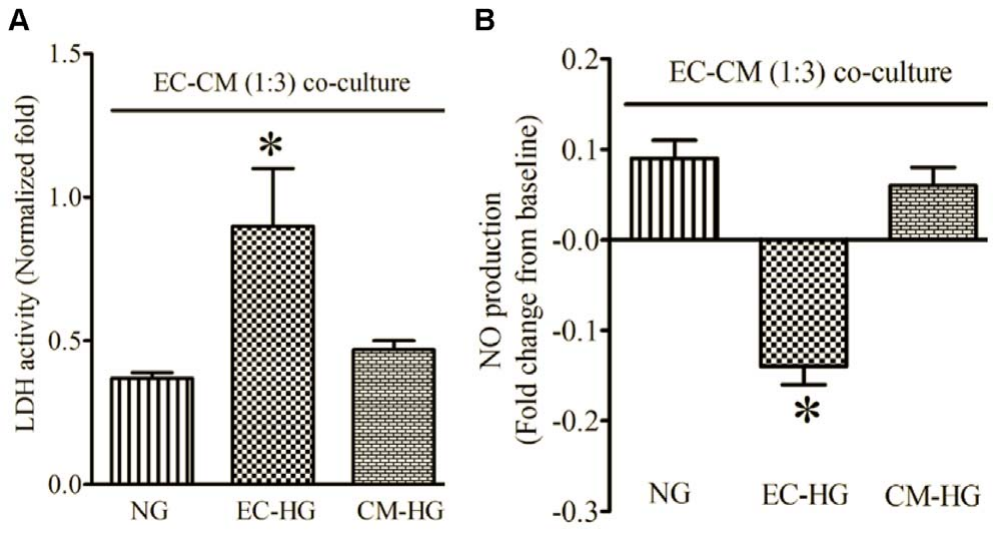
Hyperglycemia blocked endothelial cell (EC)-derived protection of cardiomyocytes (CMs) subjected to hypoxia and reoxygenation injury in EC-CM co-culture. (A) LDH activity normalized to CMs alone; (B) NO production expressed as fold change from baseline. ECs and CMs were precultured in normal or high glucose media for 12 h and subsequently cultured together. NG, endothelial cells and cardiomyocytes precultured in normal glucose media; EC-HG, endothelial cells precultured in high glucose media; CM-HG, cardiomyocytes precultured in high glucose media. *P < 0.05 vs. NG (n = 6 dishes/group).

H/R had no effect on LDH activity of ECs (data not shown). Co-culture of ECs and CMs at ratios of 1∶12 and 1∶6 did not significantly alter LDH activity following H/R injury compared with CMs alone (Figure 4A). Increasing the ratio of ECs to CMs to 1∶3 resulted in a significant decrease in LDH activity after H/R. NO production was significantly increased after H/R in co-cultured ECs and CMs compared with CMs alone (Figure 4B). The effects of ECs on CMs (LDH activity and NO production) were blocked by L-NMMA. Culture of ECs, but not CMs, in high glucose media also blocked EC-derived protection against H/R injury (Figure 5A) and NO production (Figure 5B).

### 3. SEP Restored EC-derived Protection of CMs during HG by eNOS Dimerization

**Figure 6:**
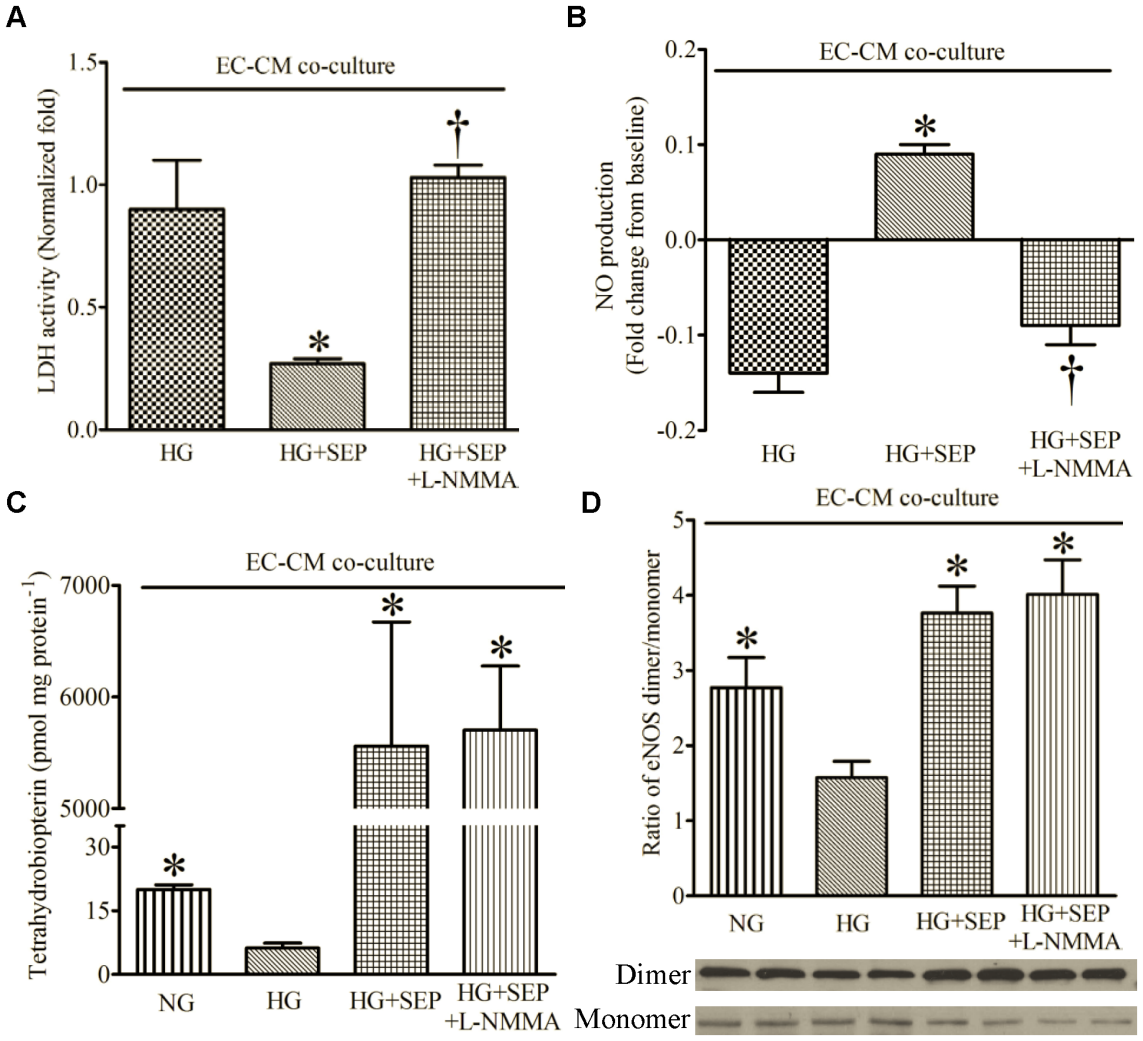
Sepiapterin (SEP) decreased LDH activity and increased NO production, tetrahydrobiopterin, and the ratio of eNOS dimer/monomer in the presence of high glucose (HG) after hypoxia and reoxygenation. (A) LDH activity normalized to cardiomyocytes (CMs) alone; (B) NO production; (C) tetrahydrobiopterin; (D) ratio of eNOS dimer/monomer [bottom: representative Western blot bands of eNOS dimer and monomer from the extract of co-cultured CMs and endothelial cells (ECs)]. NG = normal glucose media; L-NMMA = L-N^G^-monomethyl arginine. *P < 0.05 vs. HG; ^†^P < 0.05 vs. HG+SEP (n = 4–9 dishes/group).

SEP restored the productive effects of ECs on CMs during HG after H/R (Figure 6A), concomitantly with increased NO (Figure 6B) and BH_4_ concentrations (Figure 6C) compared with HG alone (n = 6, P < 0.05). As expected, L-NMMA blocked the beneficial effects of SEP on LDH activity and NO production, but not BH_4_ concentrations. HG significantly decreased the ratio of eNOS dimer/monomer (Figure 6D) compared with NG group (n = 6, P < 0.05), and this adverse action was abolished by SEP (n = 4, P < 0.05).

### 4. GTPCH-1 Overexpression in ECs Restored EC-derived Protection of CMs during HG by Preventing eNOS Uncoupling

**Figure 7:**
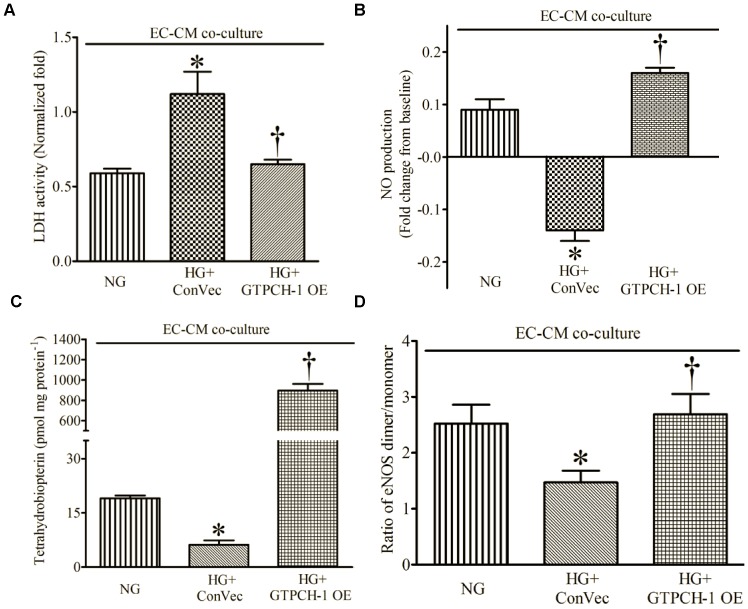
GTPCH-1 overexpression (GTPCH-1 OE) in endothelial cells (ECs) decreased LDH activity and increased NO production, tetrahydrobiopterin, and the ratio of eNOS dimer/monomer in the presence of hyperglycemia (HG) after hypoxia and reoxygenation. (A) LDH activity [relative to cardiomyocytes (CMs) alone]; (B) NO production; (C) tetrahydrobiopterin; (D) ratio of eNOS dimer/monomer. NG = normal glucose media; ConVec = control vector. *P < 0.05 vs. HG; ^†^P < 0.05 vs. HG+ConVec (n = 4–6 dishes/group).

EC infection with lentiviral control vector (ConVec) did not alter the expression of GTPCH-1 protein (data not shown). Treatment of ECs expressing ConVec with HG significantly increased LDH activity and decreased BH_4_ concentrations, NO production, and the ratio of eNOS dimer/monomer compared with ECs and CMs co-cultured in normoglycemic medium (Figure 7). Overexpression of GTPCH-1 in ECs reversed the deleterious effects of HG on cell injury, NO production, and BH_4_ concentrations after H/R. In addition, the ratio of eNOS dimer/monomer was restored by GTPCH-1 overexpression during HG.

### 5. SEP Supplementation and GTPCH-1 Overexpression Increased eNOS Phosphorylation

**Figure 8:**
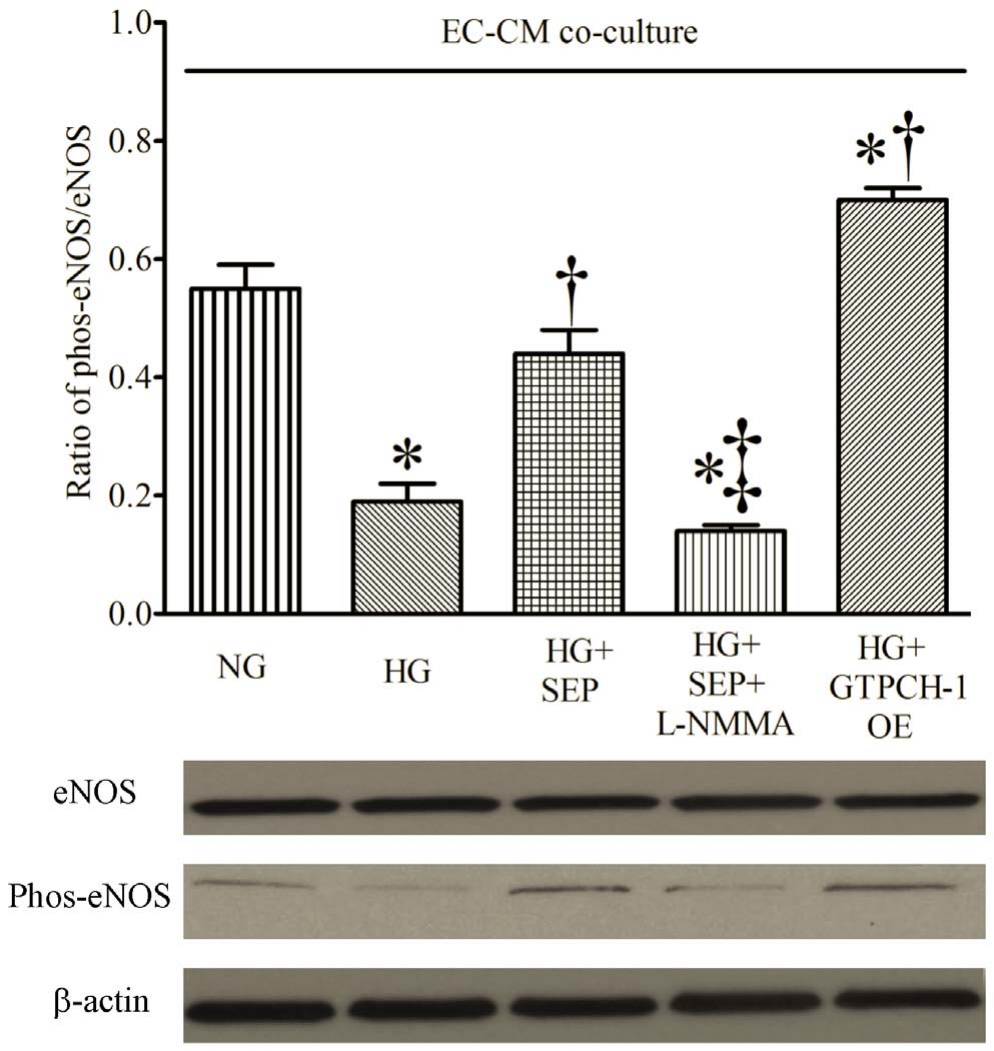
Sepiapterin (SEP) and GTPCH-1 overexpression (GTPCH-1 OE) in endothelial cells increased eNOS phosphorylation in endothelial cell-cardiomyocyte co-culture (EC-CM co-culture) undergoing hypoxia and reoxygenation injury during high glucose (HG). Top: densitometry analysis showing the ratio of phosphorylated eNOS (phos-eNOS) to total NOS; bottom: representative Western blotting bands. *P < 0.05 vs. NG; ^†^P < 0.05 vs. HG; ^‡^P < 0.05 vs. HG+SEP (n = 6 dishes/group).

The ratio of phos-eNOS to total eNOS was significantly decreased by HG in EC-CM co-culture (Figure 8). However, this detrimental effect of HG was reduced by either SEP or GTPCH-1 overexpression in ECs. The beneficial actions of SEP on activation of eNOS were blocked by L-NMMA.

## Discussion

The results of the present investigation demonstrate that ECs exert protective actions on CMs during H/R via eNOS-derived NO, and that HG abolishes the protective effect of ECs on CMs. Intriguingly, increased BH_4_ content in ECs by pharmacological and genetic approaches restores the protective effect of ECs on CMs during HG. Thus, BH_4_ is likely to play a key role in maintaining the physiological function of eNOS and eNOS-derived NO production during H/R.

The endothelium forms a metabolically active lining of blood vessels and plays a major role in regulating blood flow by altering underlying vascular smooth muscle tone. [36], [37] In the present study, a bolus injection of TX-100 in Langendorff-perfused mouse hearts abolished the vasodilatory response to the endothelium-dependent vasodilator Ach, but not to the endothelium-independent vasodilator NP. TX-100 treatment resulted in coronary endothelial dysfunction, but did not change vascular smooth muscle responsiveness to NP. TX-100-treated hearts also displayed more profound decreases in ventricular function after I/R compared with control hearts. In the EC-CM co-culture model, addition of ECs to CMs at a ratio of 1∶3 significantly decreased CM injury after H/R, and this action was blocked by HG. Taken together, the results suggest a critical role of the endothelium to modulate recovery from I/R injury.

We used the 1∶3 ratio of EC to CM in the EC-CM co-culture. In mammalian hearts, the ratios of EC to CM are 2–4∶1. [38] To enhance the ratio of EC to CM to 3∶1, we added more ECs to the co-culture system, which resulted in the death of more ECs (data not shown). It is likely that cultured ECs were heavier than those in native myocardium, therefore clumping together and lacking adhesion during the co-culture process. [39] Enhanced ratios of EC to CM by decreasing CMs caused significant amounts of cell death in the control group (CM alone). It is likely that limited cell-cell communication between CMs during the seeding process results in the death of more CMs.

In the healthy heart, CMs are intimately related to ECs in a web-like network of capillaries. [38] Communication between ECs and CMs is important in maintaining physiological regulation of CMs. [29], [38], [40] eNOS is constitutively expressed in ECs, [41] and eNOS-derived NO serves as a pivotal endothelium-derived modulator that maintains normal function of the vasculature. [42], [43] Short episodes of myocardial I/R (ischemic preconditioning) have previously been shown to increase the release of NO from the endothelium. [22], [23] To investigate whether EC-derived NO contributes to CM protection, co-culture experiments were performed in the presence of L-NMMA, a non-specific inhibitor of NOS. EC-derived cardioprotection of CMs against H/R injury was blocked by L-NMMA, and this occurred concomitantly with a decrease in EC-derived NO.

HG has previously been shown to have a major impact on mortality after myocardial infarction, [2] and HG is an independent predictor of cardiovascular morbidity and mortality. [44] Endothelial dysfunction is regarded as one of the most significant contributors to the development of vascular lesions during diabetes mellitus. [3], [5] In the present study, exposure of ECs but not CMs to high glucose media abolished EC-derived CM protection against H/R injury in the co-culture model. This was accompanied by a reduced concentration of endothelial BH_4_ and NO bioavailability. The findings suggest that decreased NO bioavailability caused by HG contributes to endothelial dysfunction, [45], [46] and that this endothelial defect negatively impacts survival of CMs following H/R. Endothelial BH_4_ is important for eNOS activity. [47], [48] However, during HG, BH_4_ is oxidized to enzymatically incompetent dihydrobiopterin due to increased production of reactive oxygen species and peroxynitrite, ultimately leading to eNOS uncoupling and production of superoxide in lieu of NO. [24], [25] Thus, it is likely that biopterin metabolism plays a central role in HG-elicited eNOS dysfunction.

BH_4_ is synthesized through two distinct pathways: the *de novo* and salvage pathways. [49], [50] GTPCH-1 is the first and rate-limiting enzyme in the *de novo* pathway, catalyzing the BH_4_ formation from GTP via enzymatic reactions. In ECs, the expression and activity of GTPCH-1 determine BH_4_ levels. [51] Alternatively, the salvage pathway enzyme dihydrofolate reductase converts SEP to BH_4_. [52] In the present study, both pharmacological (SEP) and genetic (overexpression of GTPCH-1) strategies impacting both pathways were used to increase endothelial BH_4_ concentrations, NO production, and reduce H/R injury during HG. Thus, both GTPCH-1 and BH_4_ may serve as therapeutic targets for CM protection during H/R in the presence of HG.

Culturing ECs in hyperglycemic medium for 12 h decreased the ratio of eNOS dimer/monomer and caused endothelial dysfunction in the co-culture model. Previous evidence indicates that only the dimeric form of eNOS is enzymatically active and able to generate NO. [53] In contrast, the monomeric form of the enzyme is a marker for uncoupling of eNOS, during which transfer of electrons from the reductase to the oxygenase domains is not coupled to L-arginine oxidation. The latter contributes to superoxide formation with subsequent deleterious effects. It is likely that monomerization of eNOS elicited by HG contributes to endothelial dysfunction. Intriguingly, both administration of SEP and GTPCH-1 overexpression in ECs dramatically increased BH_4_ content and restored the ratio of eNOS dimer/monomer and phos-eNOS/total eNOS during HG. The results suggest that BH_4_ may enhance the stabilization and activity of eNOS in the presence of HG. [47], [48].

The present study indicates HG increases the vulnerability of CMs to H/R injury by a BH_4_/eNOS/NO pathway-dependent mechanism. This conclusion should be interpreted within the constraints of potential limitations. Neuregulin-1 is synthesized as a transmembrane protein in myocardial microvascular ECs and binds to ErbB receptors on CMs to regulate the function of CMs. [54], [55] Previous studies indicate that neuregulin-1 increases eNOS phosphorylation and NO production and protects CMs from apoptotic cell death produced by oxidative stress.[55]–[57] Recent evidence suggests that the neuregulin-1/ErbB signaling pathway is impaired in diabetic myocardium. [58] It remains unclear whether the neuregulin-1/ErbB signaling pathway is involved in the detrimental effect of HG on endothelial-myocardial interaction.

Exposure of ECs to HG attenuated the protective effect of ECs on CMs subjected to H/R injury in cultured cells. To examine the detrimental effect of HG on endothelial function in intact animals, C57BL/6 mice were injected intraperitoneally with 2 g/kg dextrose for 5 consecutive days to induce acute HG. Despite the high mortality in the dextrose-treated mice, coronary artery isolated from the surviving mice had significant relaxation responses to Ach (data not shown). These results suggest that coronary endothelial function is preserved in the dextrose-treated mice. It may be in part related to an increase in insulin, [59] that produces the vasodilatory effect by the stimulation of the release of NO. [60] To eliminate the confounding response of insulin, we injected streptozotocin 200 mg/kg into C57BL/6 mice to induce total insulin deficiency. Although blood glucose was higher than 350 mg/dl 2 weeks after streptozocin injection, isolated coronary artery displayed marked relaxation responses to Ach 4 weeks after the streptozotocin injection. Further experiments with prolonged period of HG will be performed to study the detrimental effect of HG on coronary endothelial function *in vivo*.

In summary, HG-induced decreases in coronary endothelial BH_4_ exacerbate the vulnerability of myocardium to I/R injury by reducing eNOS-derived NO. Enhanced BH_4_ content in ECs can increase CM tolerance to ischemic stress during HG. Thus, preservation of sufficient endothelial BH_4_ during I/R could be an effective strategy for cardioprotection during HG or diabetes.
